# Isolated Bilateral Foveal Hypoplasia Diagnosed in Adulthood Following Evaluation for Presbyopia

**DOI:** 10.7759/cureus.112263

**Published:** 2026-07-08

**Authors:** Houda Safwate, Hala Ait Amar, Amine Razzak, Mohamed Bouazza, Mohamed Elbelhadji

**Affiliations:** 1 Department of Ophthalmology, Faculty of Medicine, Mohammed VI University of Health Sciences (UM6SS), Casablanca, MAR

**Keywords:** fluorescein angiography, foveal hypoplasia, fovea plana, nystagmus, optical coherence tomography

## Abstract

Foveal hypoplasia, also known as fovea plana, is an uncommon congenital anomaly caused by incomplete foveal differentiation during ocular development. Although it is usually diagnosed in childhood, particularly in patients with associated ocular or systemic disorders, isolated forms may remain unrecognized until adulthood. We report the case of a 45-year-old woman who presented for the first time with persistent near-vision difficulty, initially attributed to presbyopia. Despite appropriate refractive correction, her visual symptoms did not fully improve. Ophthalmic examination revealed bilaterally reduced best-corrected visual acuity (20/40 in the right eye and 20/32 in the left eye) and low-amplitude horizontal nystagmus. Color fundus photography showed the absence of the foveal reflex in both eyes. Fluorescein angiography demonstrated loss of the physiologic foveal dark spot with a qualitatively reduced foveal avascular zone. Spectral-domain optical coherence tomography (SD-OCT) confirmed bilateral fovea plana, showing absence of the foveal pit, persistence of the inner retinal layers at the foveal center, increased central retinal thickness, and preserved outer retinal bands, with no evidence of acquired macular disease. No syndromic or systemic association was identified. This case highlights that isolated foveal hypoplasia may remain undiagnosed until adulthood and may be detected when presbyopia unmasks long-standing visual limitations. SD-OCT is essential for diagnosis, while fluorescein angiography provides supportive vascular findings and helps distinguish this congenital condition from acquired macular disease.

## Introduction

The fovea is the central retinal specialization responsible for high-resolution central and color vision, largely because of the dense specialization of cone photoreceptors and displacement of the inner retinal layers [1,2]. Its formation is one of the most precisely coordinated events in ocular development. From mid-gestation onward, the inner retinal layers, including ganglion cells and bipolar cells, gradually migrate centrifugally away from the foveal center, while cone photoreceptors progressively specialize in this region. The foveal pit is the anatomical result of this process, and its absence reflects incomplete foveal differentiation [1,2].

Foveal hypoplasia, also referred to as “fovea plana,” results from incomplete development of the foveal pit and persistence of the inner retinal layers at the foveal center [2,3]. It is most commonly recognized in association with ocular or systemic conditions such as oculocutaneous albinism, aniridia, nanophthalmos, and achromatopsia, in which reduced visual acuity and nystagmus often prompt evaluation early in life [2,4]. The isolated form, however, is less frequently reported and may be overlooked, particularly when visual impairment is mild and no syndromic features are present [5,6].

This case highlights the importance of considering isolated foveal hypoplasia in adults with unexplained reduced visual acuity or an incomplete response to refractive correction. We present a 45-year-old woman in whom bilateral isolated fovea plana was diagnosed for the first time after evaluation for persistent near-vision difficulty, initially attributed to presbyopia.

## Case presentation

A 45-year-old stay-at-home mother was referred to our ophthalmology department for persistent near-vision difficulty that had not resolved with reading glasses. She had no previous diagnosis of ocular disease and no history of ocular inflammation, uveitis, transient central visual loss suggestive of central serous chorioretinopathy, ocular trauma, ocular surgery, corticosteroid exposure, or medication use associated with macular disease. She had no known systemic illness and no family history of visual impairment, nystagmus, albinism, or inherited retinal disease.

On examination, best-corrected distance visual acuity was 20/40 in the right eye and 20/32 in the left eye. Pinhole testing did not produce any measurable improvement. Slit-lamp examination was normal in both eyes, with clear corneas, quiet anterior chambers, normally configured irides without transillumination defects, and clear lenses. Intraocular pressure was within the normal range. Ocular motility examination revealed a low-amplitude, right-beating horizontal nystagmus in primary gaze, with no clear null point and no manifest strabismus.

**Figure 1:**
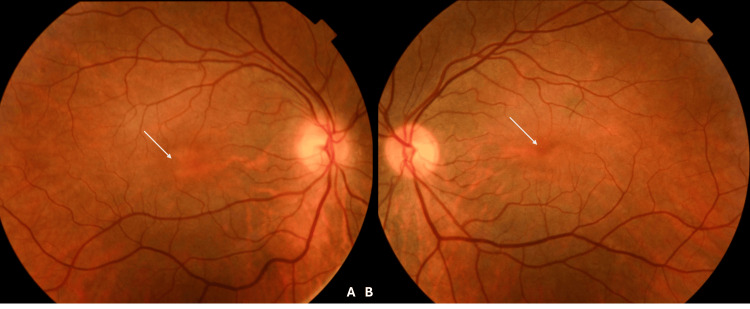
Color fundus photographs of the right (A) and left (B) eyes showing the absence of the foveal light reflex (white arrows).

Dilated fundus examination revealed healthy optic discs with sharp margins and a cup-to-disc ratio of approximately 0.3 in each eye. The retinal vasculature appeared normal in caliber, course, and branching pattern, and the peripheral retina was unremarkable. Examination of the macular region disclosed a dull, grayish appearance with an abnormal pigmentary pattern and complete absence of the foveal light reflex in both eyes. No epiretinal membrane, drusen, macular edema, or signs of acquired maculopathy were identified (Figure 1).

**Figure 2:**
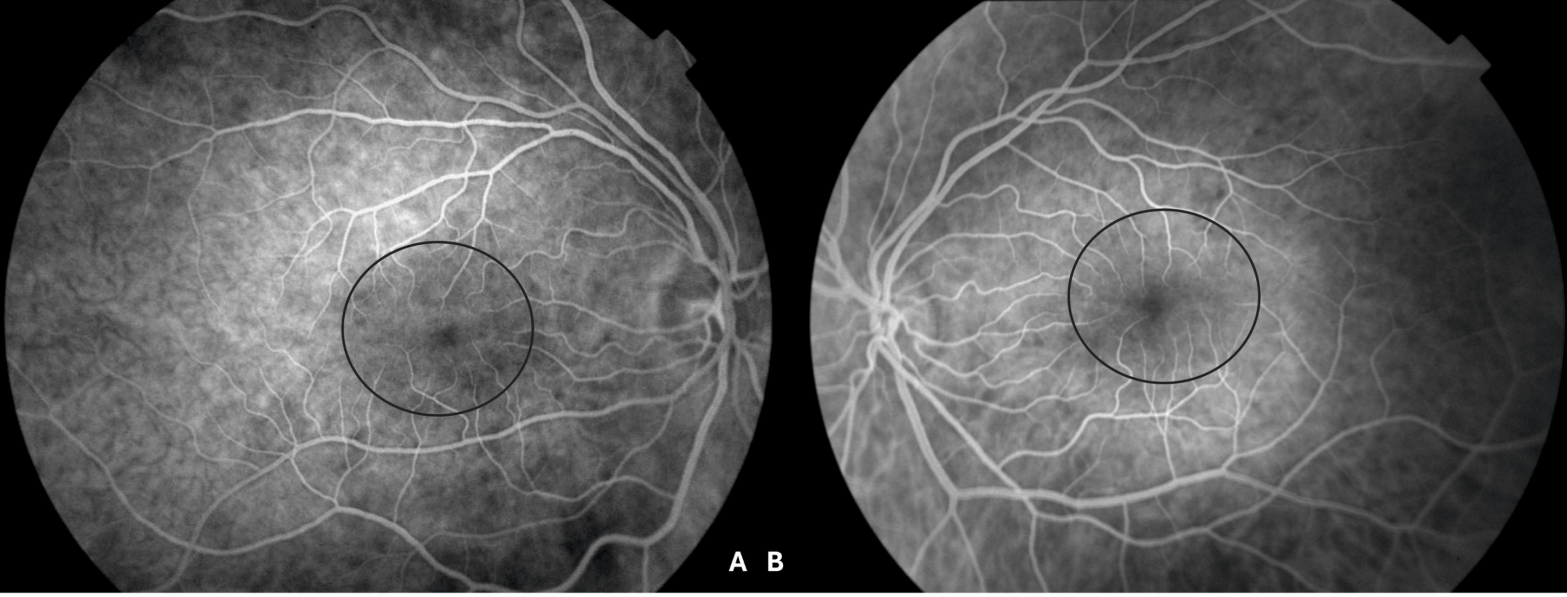
Fluorescein angiography of the right (A) and left (B) eyes showing a reduced foveal avascular zone. Open circles indicate the qualitatively reduced foveal avascular zone, with retinal vessels extending abnormally close to the foveal center in both eyes and loss of the normal foveal dark spot configuration.

Fluorescein angiography provided supportive vascular evidence. In both eyes, the early arteriovenous phase showed attenuation of the physiologic foveal hypofluorescence, with loss of the well-defined foveal dark spot normally seen in a healthy macula. The foveal avascular zone was qualitatively reduced in both eyes, with retinal vessels extending abnormally close to the foveal center, a pattern supporting a congenital abnormality of foveal neurovascular development. The remainder of the angiogram showed no leakage, neovascularization, or late staining in either eye (Figure 2).

**Figure 3:**
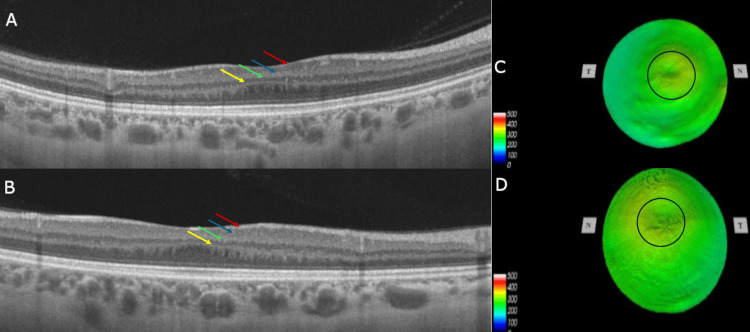
SD-OCT B-scans and retinal thickness maps of the right eye (A, C) and left eye (B, D). B-scans of the right eye (A) and left eye (B) show the absence of the foveal pit with a flat macular profile. The nerve fiber layer (red arrow), ganglion cell layer (blue arrow), inner plexiform layer (green arrow), and inner nuclear layer (yellow arrow) all persist at the foveal center, where they should normally be absent. Retinal thickness maps (C, D) show increased central thickness, with no central depression and absence of the expected central blue zone normally corresponding to the thin foveal floor (black circle), supporting the diagnosis of bilateral foveal hypoplasia. SD-OCT, spectral-domain optical coherence tomography

Spectral-domain optical coherence tomography (SD-OCT) provided the definitive structural findings (Figure 3A, 3B). Radial macular B-scans through the foveal center demonstrated complete absence of the foveal pit, with a flat macular contour in both eyes. Retinal thickness mapping using the device’s automated ILM-to-OS/RPE segmentation confirmed increased central retinal thickness, measuring 286 µm in the right eye and 300 µm in the left eye. These values are higher than the typical foveal thickness reported in normal eyes (approximately 150-200 µm), with no visible central thinning pattern on the thickness maps (Figure 3C, 3D). Layer-by-layer assessment showed persistence of the inner retinal layers at the foveal center in both eyes, where these layers would normally be absent. The ellipsoid zone and external limiting membrane were preserved and continuous throughout both scans. No intraretinal fluid, serous detachment, epiretinal membrane, macular hole, or neovascular tissue was detected in either eye.

The angiographic findings supported the OCT diagnosis by showing a qualitatively reduced foveal avascular zone, with retinal vessels extending abnormally close to the foveal center. The combination of a flat macular profile, increased central retinal thickness, persistent inner retinal layers at the foveal center, and preserved outer retinal bands was consistent with bilateral foveal hypoplasia, also referred to as fovea plana. Because the available OCT images did not allow reliable assessment of all numerical grade-defining features, including outer segment lengthening and outer nuclear layer widening, no specific Thomas grade was assigned. No clinical feature raised suspicion for albinism, aniridia, or another recognizable associated syndrome. The diagnosis of isolated bilateral foveal hypoplasia was established for the first time at the age of 45.

## Discussion

This case is notable because isolated bilateral foveal hypoplasia remained unrecognized until adulthood and was identified only after incomplete improvement of near-vision symptoms with presbyopic correction. Although foveal hypoplasia is a well-characterized congenital entity, its isolated form may be overlooked when visual impairment is mild, bilateral, and not accompanied by obvious syndromic features [3-6].

Presbyopia may have contributed to the recognition of long-standing visual limitations in this patient. With the age-related decline in accommodative amplitude, visual demands for near work may have exceeded the patient’s preexisting functional reserve. The incomplete response to reading correction, therefore, suggested that the symptoms were not purely refractive and prompted further ophthalmologic investigation.

The absence of associated ocular or systemic features meant that no contextual clue initially directed suspicion toward a congenital macular anomaly. In such cases, SD-OCT is essential for diagnosis [5,6]. This is consistent with recent observations by Lejoyeux et al., who reported fovea plana incidentally in adults undergoing preoperative cataract evaluation and in fellow eyes of patients assessed for epiretinal membrane, suggesting that this condition may be more frequently encountered in adults than previously thought [7,8].

In adults, isolated foveal hypoplasia should be differentiated from subtle macular dystrophies, sequelae of central serous chorioretinopathy or resolved macular edema, epiretinal membrane, and other acquired maculopathies. In this case, bilateral symmetry, absence of exudative or tractional macular changes, persistent inner retinal layers with an absent foveal pit on SD-OCT, preserved outer retinal bands, and a reduced foveal avascular zone favored a congenital developmental anomaly over acquired macular disease [3-6,9,10]. The absence of iris transillumination defects, aniridia, hypopigmentation, systemic disease, or a family history supported the isolated form [2,4-6].

Each imaging modality contributed distinct information. Fundus photography identified the absent foveal reflex but remained nonspecific. Fluorescein angiography provided supportive vascular evidence by demonstrating attenuation of the foveal dark spot and a qualitatively reduced foveal avascular zone, findings that favored a developmental abnormality rather than an acquired macular process [9,10]. SD-OCT established the definitive diagnosis by revealing persistent inner retinal layers at the foveal center and, equally importantly, excluding the hallmarks of acquired macular pathology. Although optical coherence tomography angiography was not performed in our patient, recent studies using this modality have demonstrated altered foveal avascular zone morphology and increased foveal flow density in eyes with fovea plana, further reinforcing the diagnostic value of foveal microvascular assessment [10,11].

The preserved outer retinal bands support classification within the typical form of foveal hypoplasia rather than the atypical form characterized by photoreceptor band disruption [4]. However, because the available OCT images did not permit reliable assessment of all numerical grade-defining features, including outer segment lengthening and outer nuclear layer widening, a specific Thomas grade was not assigned [4]. Functionally, the preserved outer retinal structure is consistent with the relatively mild bilateral reduction in visual acuity observed in this patient. The low-amplitude nystagmus may reflect fixation instability related to incomplete foveal specialization, although this finding is not always prominent in mild isolated cases [1,2]. No treatment can currently restore normal foveal architecture. Management in this patient has, therefore, focused on optimizing refractive correction, explaining the congenital and nonprogressive nature of the condition, and scheduling periodic follow-ups to monitor for any change in visual acuity over time.

## Conclusions

Isolated foveal hypoplasia may remain undetected until adulthood and can mimic refractive or age-related visual complaints, including presbyopia. It should be considered in adults with unexplained reduced visual acuity, an absent foveal reflex, or mild nystagmus, even in the absence of syndromic features.

SD-OCT is the key diagnostic examination, demonstrating the absence of the foveal pit and the persistence of the inner retinal layers at the foveal center. Fluorescein angiography can provide supportive vascular findings, including reduction of the foveal avascular zone. Recognition of this congenital and nonprogressive condition is important to avoid misdiagnosis, unnecessary investigations, and inappropriate treatment.
